# Life course and intrinsic ability in Brazilian adults and older adults: a mediation analysis

**DOI:** 10.11606/s1518-8787.2026060007512

**Published:** 2026-10-19

**Authors:** Milara Barp Dias, Flávia Silva Arbex Borim, Daniella Pires Nunes, Isabella Amorim de Araújo Pereira, Valéria Pagotto

**Affiliations:** I Universidade Federal de Goiás Programa de Pós-Graduação em Enfermagem e Saúde Goiânia GO Brasil Universidade Federal de Goiás. Programa de Pós-Graduação em Enfermagem e Saúde. Goiânia, GO, Brasil; II Universidade Estadual de Campinas Programa de Pós-Graduação em Gerontologia Campinas SP Brasil Universidade Estadual de Campinas. Programa de Pós-Graduação em Gerontologia. Campinas, SP, Brasil; III Universidade Cidade de São Paulo Programa de Pós-Graduação em Fisioterapia São Paulo SP Brasil Universidade Cidade de São Paulo. Programa de Pós-Graduação em Fisioterapia. São Paulo, SP, Brasil; IV Universidade Estadual de Campinas Programa de Pós-Graduação em Enfermagem Campinas SP Brasil Universidade Estadual de Campinas. Programa de Pós-Graduação em Enfermagem. Campinas, SP, Brasil

**Keywords:** Life Course, Intrinsic Capacity, Adults, Older Adults, Aging

## Abstract

**OBJECTIVE:**

To analyze the direct and indirect associations between adverse life course conditions and impairment of intrinsic capacity in Brazilians aged 50 years or older.

**METHODS:**

An analytical cross-sectional study using data from the second wave of the Estudo Longitudinal da Saúde dos Idosos Brasileiros (ELSI-Brasil) (n = 4,817). Intrinsic capacity was assessed across five domains (cognition, vitality, mobility, psychological aspects, and sensory aspects) and was considered impaired when three or more domains were affected. Childhood adversities included parental illiteracy, lack of running water at home, and food insecurity. Educational attainment and income in adulthood were considered mediating variables. Path analysis was used.

**RESULTS:**

Childhood adversities had significant direct and indirect effects on the impairment of intrinsic capacity. These effects were mediated by lower educational attainment and income in adulthood. Educational attainment stood out as a central mediator, exerting a direct protective effect on intrinsic capacity and an indirect effect through its influence on income.

**CONCLUSIONS:**

The findings reinforce the relevance of the life-course perspective for understanding inequalities in aging and indicate educational attainment as a priority target for interventions aimed at promoting healthy aging in the Brazilian context.

## INTRODUCTION

Intrinsic capacity (IC) is defined as the set of physical and mental attributes of a person that interact with the environment and that, when maintained and optimized throughout life, promote healthy aging. Proposed in 2019 based on the English Longitudinal Study on Ageing (ELSA), the concept assesses five domains: mobility, vitality, cognition, psychological aspects, and sensory functions. Although it is a pillar of the “Decade of Healthy Aging (2021–2030),” evidence on IC in the Brazilian context remains scarce, especially regarding an understanding of how life trajectories in Brazil, marked by historical inequalities, impact these domains in an integrated manner. This gap limits the development of specific strategies that consider the heterogeneity of aging in Brazil^1^.

This comprehensive approach to the health of older adults reflects an important conceptual advance considering the realization that conceptions of aging centered exclusively on diseases or disabilities are insufficient to capture the complexity of people’s lives and health as they age^1^. While functionality generally focuses on what a person *is able to do* (such as performing activities of daily living), IC focuses on what a person possesses in terms of biological and psychological reserves and potential. Previous studies have identified sociodemographic and health factors associated with IC, including sex^2^, age^3^, income level^4^, marital status^4^, educational level^5^, behaviorshealth-related^5^, and the burden of chronic diseases^3,4^.

Despite the relevance of these studies, analyses conducted at a single point in time may not fully capture the factors that contribute to the occurrence of IC, given that each person’s life course varies over time and is influenced by individual, social, and contextual factors.

Given this, studying the IC from a life-course perspective is essential to understanding how experiences, exposures, and contexts encountered throughout an individual’s life course influence aging and health in later life, considering that aging is a multidetermined, continuous, and dynamic process. Life-course models theorize that events during critical periods of human development and the accumulation of risks throughout life influence health, disease, and aging^6,7^. Adversities in childhood, such as low socioeconomic status^8^ and food insecurity^9^, are associated with the development of physical diseases^10^ and mental disorders in adulthood^11^.

The relationship between life course variables and IC has already been documented^12,13^. Childhood circumstances such as low parental education, poorer childhood health, and living in substandard housing were directly associated with lower levels of IC in later life, with part of this effect mediated by accumulated socioeconomic inequalities^13^. Furthermore, the areas of IC most affected by these conditions are primarily the cognitive, sensory, and psychological domains^12^.

Evidence on the association between life course trajectories and impaired IC remains scarce in the Brazilian context, particularly when considering all its domains. Furthermore, this approach facilitates advances in scientific knowledge by integrating biological, psychological, and social perspectives, thereby enriching our understanding of the aging process.

In light of this, the objective of the study was to analyze the direct and indirect association between adverse life course conditions and impaired IC in a Brazilian population of adults aged 50 years or older, using baseline data from the Estudo Longitudinal da Saúde dos Idosos Brasileiros (ELSI-Brasil – Longitudinal Study on the Health of Brazilian Older Adults).

## METHODS

### Study Design and Population

This was a cross-sectional, analytical study based on data from the second wave of ELSI-Brasil, conducted between 2019 and 2021 (Figure 1). ELSI-Brasil is a national, household-based cohort representative of the Brazilian population aged 50 years or older living in the community, whose objective is to investigate the aging process and its determinants, thereby supporting the formulation and evaluation of public policies in the country^14^.

**Figure 1:**
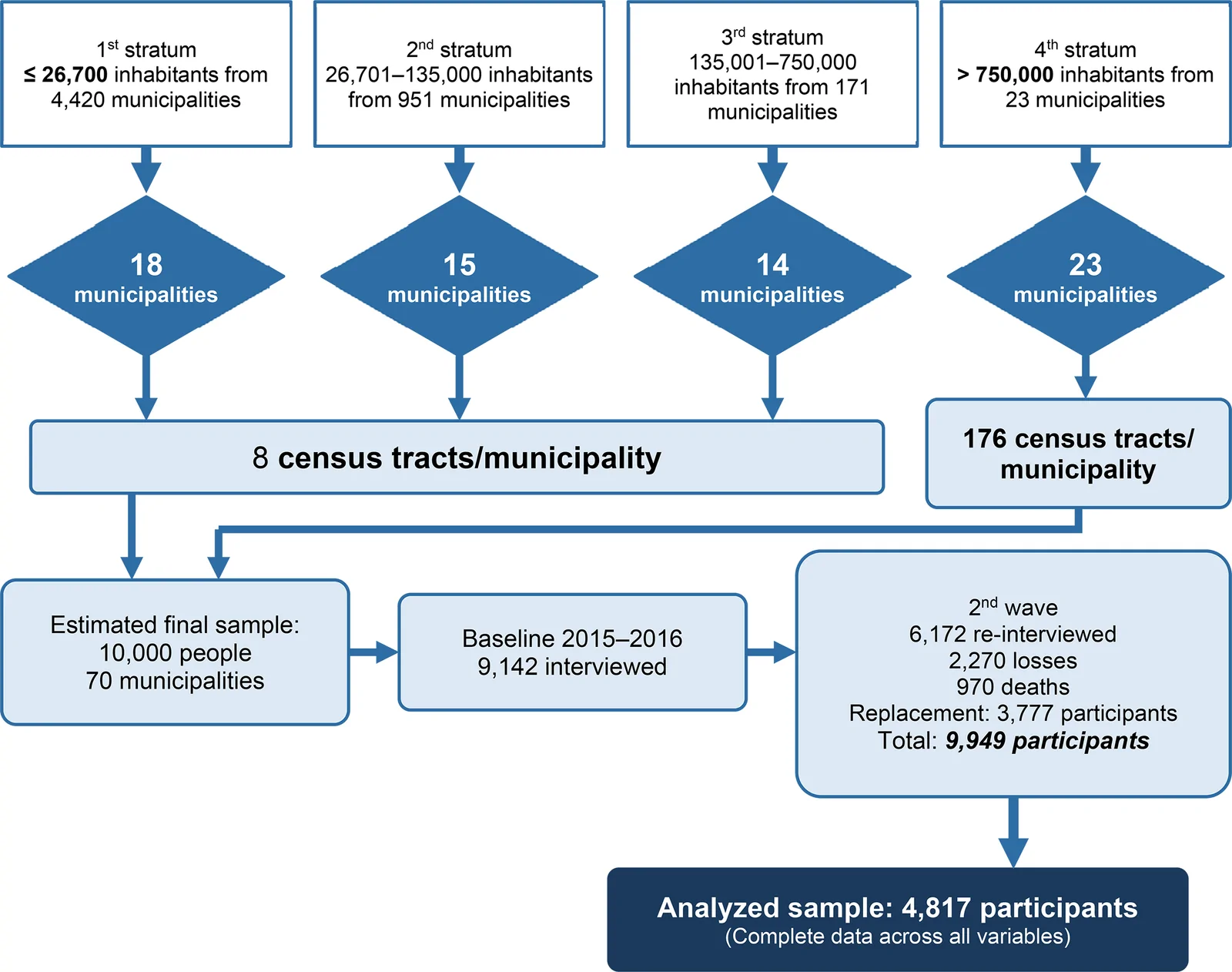
Summary flowchart of the sampling process. ELSI-Brasil 2019–2021.

### Study Sample and Sampling

The ELSI-Brasil sampling plan is complex, probabilistic, and multistage, involving the selection of municipalities, census tracts, and households. The design ensures national representativeness, covering urban and rural areas in 70 municipalities distributed across Brazil’s five regions (Figure 1).

The baseline phase of the study was conducted between 2015 and 2016, with 9,412 participants. In the second wave, 6,172 individuals were re-interviewed; there were 2,270 follow-up losses and 970 deaths. To maintain the cohort’s age representativeness, 3,777 new participants were included, bringing the total number of individuals interviewed in the second wave to 9,949^14^ (Figure 1).

For this study, only cross-sectional data from the second wave were used. All residents of the selected households aged 50 years or older were eligible. The final analytical sample consisted of 4,817 individuals, including only those participants with complete information for all variables of interest (Figure 1).

### Data Collection

Data collection was performed by previously trained interviewers using standardized questionnaires, in addition to physical tests and laboratory exams, following protocols previously described in the ELSI-Brasil study^14^.

### Study Variables

#### Dependent Variable

The variable of interest was IC, assessed based on five domains: cognition, psychological health, sensory function, vitality, and mobility^1^.

The cognitive domain was measured using the Verbal Fluency Test (VFT), in which participants named as many animals as possible in one minute. The cutoff scores were nine words for individuals with up to eight years of schooling and 13 for the others, indicating preserved cognition^15^.

The psychological domain was assessed using the abbreviated CES-D scale, consisting of eight items on depressive symptoms. Scores ≤ 4 indicated no impairment^16^.

Vitality was measured by handgrip strength using a dynamometer, with the average of three trials. The cutoff points were > 27 kgf for men and > 16 kgf for women, according to the criteria of the European Working Group on Sarcopenia^17^.

Locomotion was assessed using the habitual walking test over a three-meter distance, measured twice. Mean values ≥ 0.8 m/s indicated preserved function^18^.

Sensory assessment was based on self-reported visual and auditory function. Responses of “very good,” “good,” or “fair” for distance vision, near vision, and hearing indicated preserved function^3^.

Each IC domain was classified as 0 (preserved) or 1 (impaired). The sum of these scores yielded an index ranging from 0 to 5 points; IC was considered impaired if three or more domains were impaired^2^.

#### Independent Variables

The independent variables included sociodemographic factors—sex (female or male, self-reported) and age group (categorized as 50–59, 60–69, 70–79, and ≥ 80 years)—and life course variables, namely: parental illiteracy (report of illiterate father and mother)^19^, food insecurity (report of having ever gone to bed hungry or witnessed a lack of food from birth through age 15)^9^, lack of treated water (report of no running water in the home where the individual lived at age 10)^20^—all dichotomized as “yes” or “no.” Among the mediating variables in adulthood are educational attainment (complete years of schooling, categorized as: none, 1–4, 5–8, or ≥ 9 years) and monthly income (expressed in minimum wages, based on the value of R$ 1,047.67, and categorized as: < 1, 1–3, or ≥ 4 minimum wages)^13,20^.

## Data Analysis

A descriptive analysis was performed to characterize the sample, using measures of absolute and relative frequency for the variables. Percentage distributions and their respective 95% confidence intervals were estimated using the survey (svy) module, including sample weights and stratification. Structural equation modeling was performed using the SEM command with robust standard errors (vce robust).

To study the relationship between the variables of interest, based on the pre-established theoretical model (Figure 2), structural equation modeling was used via the path analysis technique. This approach serves as an extension of the regression model, allowing for the evaluation of relationships among multiple variables^21^. Using this technique, it was possible to investigate the direct and indirect connections between independent and dependent variables.

**Figure 2:**
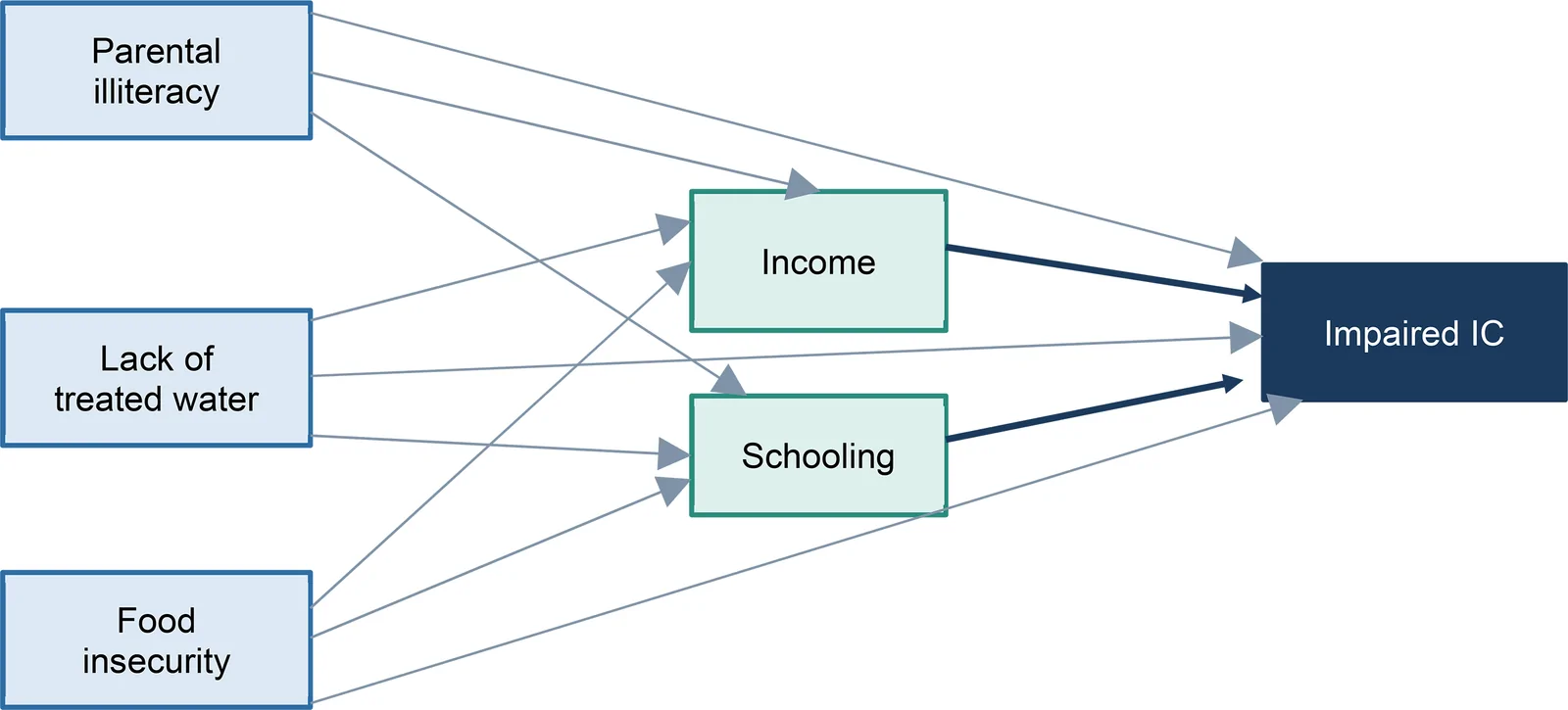
Hypothetical model of the relationship between IC and adverse life course conditions. IC: intrinsic capacity.

The direct effects of childhood conditions on IC are indicated by the arrows that directly link the independent variables to the dependent variable. The indirect effects, mediated by educational attainment and income, are represented by the arrows that pass through the mediating variables (Figure 2).

After adjusting the indicators and conducting significance tests, the final path analysis model was constructed, which retained or eliminated the relationships from the initial theoretical model. The following tests and reference values for model fit thresholds were adopted in this study: chi-square test (χ^2^) for model fit (p > 0.05), chi-square-to-degrees-of-freedom ratio (χ^2^/df) < 2, Standardized Root Mean Square Residual (SRMR) ≤ 0.10, Root Mean Square Error of Approximation (RMSEA) ≤ 0.08, Comparative Fit Index (CFI) ≥ 0.90, and Tucker–Lewis Index (TLI) ≥ 0.90.

To assess the fit of the proposed paths, significance tests were performed on the path coefficients. Absolute values of t > 1.96 were considered statistically significant. The analyses were conducted using Stata software, version 15.1.

## Ethical Considerations

The study was approved by the Ethics Committee of the Fundação Oswaldo Cruz (Fiocruz Minas – Oswaldo Cruz Foundation) (CAAE: 34649814.3.0000.5091), and all participants signed an informed consent form.

## RESULTS

The sample consisted of 4,817 participants. Of the total number of participants (n = 4,817), 54.7% were women, 50.4% were between 50 and 59 years old, and 37.9% had 1 to 4 years of schooling. Regarding life course variables, 43.7% of the older adults had illiterate parents, 64.0% experienced a lack of piped water during childhood, and 24% experienced food insecurity (Table 1).

**Table 1:** Characteristics of study participants (n = 4,817).

Variable	Total (n = 4,817)	95% CI
Gender		
Female	54.7	51.7 to 57.7
Male	45.3	42.2 to 48.3
Age group		
50–59 years	50.4	46.0 to 54.4
60–69 years	30.2	28.6 to 32.1
70–79 years	14.7	12.6 to 17.1
80 years and older	4.7	3.7 to 5.9
Education (years of schooling)		
Never attended school	11.1	8.4 to 14.6
1–4	37.9	34.7 to 41.1
5–8	21.3	19.2 to 23.4
≥ 9	29.8	26.1 to 33.6
Income (minimum wages)		
< 1	64.3	60.2 to 68.1
1–3	26.2	23.3 to 29.3
≥ 4	9.6	7.6 to 11.9
Parental illiteracy during childhood		
No	56.3	50.9 to 61.6
Yes	43.7	38.4 to 49.1
Lack of running water during childhood		
No	36.0	31.4 to 40.7
Yes	64.0	59.3 to 68.5
Childhood food insecurity		
No	76.0	72.5 to 79.3
Yes	24.0	20.7 to 27.5
Impaired intrinsic capacity		
No	73.3	68.3 to 77.7
Yes	26.7	22.3 to 31.7

95% CI: 95% confidence interval.

Figure 3 shows that both early-life variables (parental illiteracy, lack of piped water, and food insecurity) and adult-life variables (income and education) were directly associated with impaired cognitive function. It was observed that early-life variables, such as parental illiteracy (β = 0.07), lack of piped water (β = 0.03), and food insecurity (β = 0.05), were directly and positively associated with impaired CI. On the other hand, higher educational attainment (β = -0.14) and higher income (β = -0.04) showed direct protective effects. Adverse early-life conditions were also negatively associated with educational attainment (parental illiteracy: β = -0.36; lack of piped water: β = -0.24; food insecurity: β = -0.06), which, in turn, was positively associated with income in adulthood (β = 0.19). Additionally, it was observed that income and educational attainment mediated the association between early-life conditions and IC. Notably, educational attainment also acted as a mediator of the association between income and IC, as evidenced by the significant indirect effect (β = -0.01; p = 0.009) (Figure 3).

**Figure 3:**
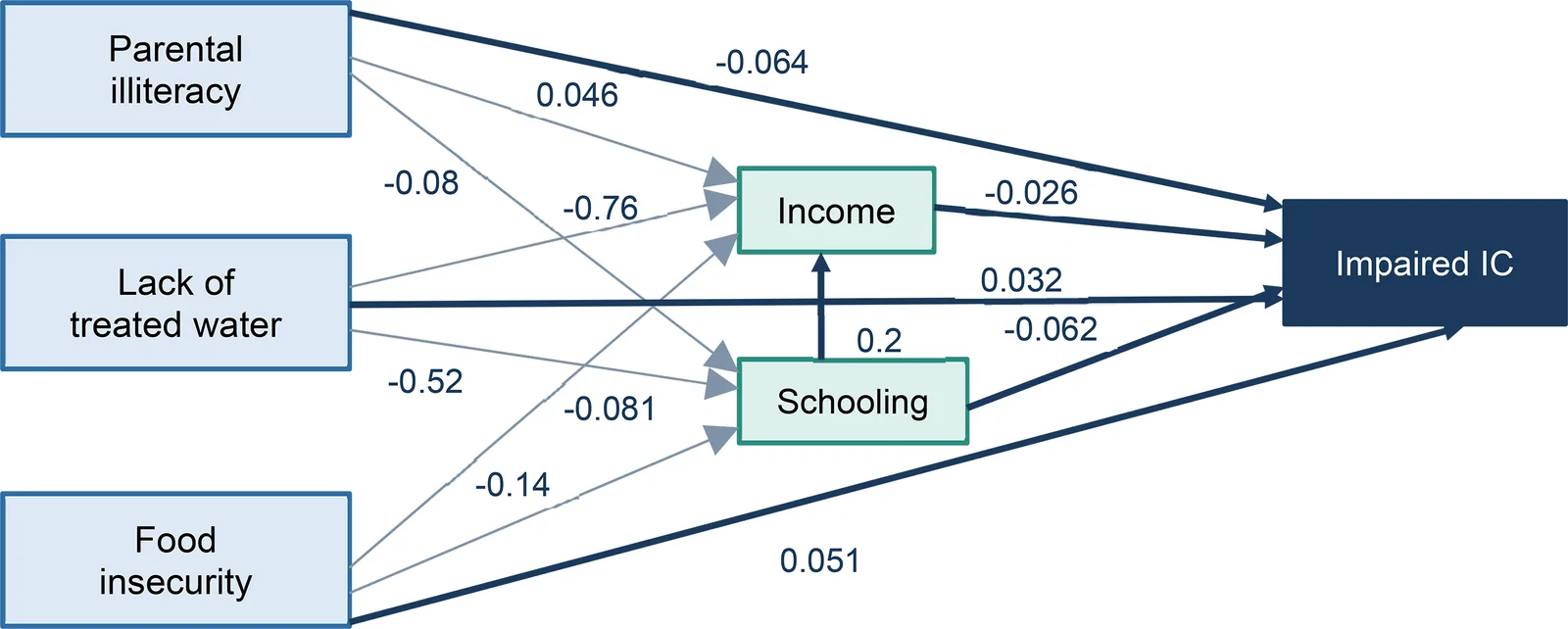
Final model of the relationship between life course variables and impaired IC, according to path analysis. IC: intrinsic capacity.

Note: The arrows represent directional associations estimated in the model, and the values correspond to the standardized coefficients (β). Thicker arrows indicate direct associations.

Table 2 presents the indirect effects, showing that all childhood conditions (parental illiteracy: β = 0.06; lack of piped water: β = 0.04; food insecurity: β = 0.01) significantly influenced impaired IC in adulthood, primarily through lower levels of education and income. Regarding adult-life variables, it is noteworthy that only educational attainment showed a protective indirect effect on IC mediated by higher income in adulthood (Table 2).

**Table 2:** Indirect effects mediated by educational attainment and income on impaired intrinsic capacity.

Indirect associations	Mediating variable	Coeff.	p > |z|	95% CI
Parental illiteracy → Impaired IC	Income	0.0521	< 0.001	0.0411 to 0.0631
Lack of running water → Impaired IC	Income	0.0368	< 0.001	0.0286 to 0.0450
Food insecurity → Impaired IC	Income	0.0117	< 0.001	0.0068 to 0.0166
Education → Impaired IC	Income	-0.0051	0.009	-0.0089 to -0.0013
Parental illiteracy → Impaired IC	Educational attainment	-0.0489	< 0.001	-0.0684 to -0.0293
Lack of running water → Impaired IC	Education	-0.0039	0.154	-0.0092 to 0.0014
Food insecurity → Impaired IC	Education	-0.0251	0.034	-0.0482 to -0.0019

Coeff.: standardized coefficients; 95% CI: 95% confidence interval; IC: intrinsic capacity.

The final structural equation model demonstrated an adequate fit to the data, as indicated by the parameters described in the data analysis (CFI = 1.00; TLI = 1.00; RMSEA < 0.001; SRMR < 0.001), suggesting a good fit of the proposed structure. Overall, the model explained 26.1% of the variation in the IC commitment.

## DISCUSSION

The results of this study showed that conditions indicative of low socioeconomic status in childhood—parental illiteracy, lack of piped water, and food insecurity—were significantly associated with impaired IC, an effect that occurred both directly and through the mediating role of lower educational attainment and income.

Although life-course evaluation is the subject of many studies, its relationship with IC has been assessed in a few recent studies^12,13^. In a general analysis of the effect of various childhood conditions on the domains of IC in adults and older adults, two studies show findings similar to ours. A cross-sectional study in China involving individuals aged ≥ 45 years showed that low parental education, poorer childhood health, and poor housing conditions were associated with lower IC, with part of this effect mediated by socioeconomic inequalities accumulated over the life course. The strongest associations were identified in the cognitive, sensory, and psychological domains of IC^13^. In an observational cohort study involving participants aged ≥ 50 years from the United States, the United Kingdom, Europe, China, and Japan, higher levels of parental education, socioeconomic status in childhood, and household income in adulthood were positively associated with IC in all analyzed cohorts^12^.

Regarding parental illiteracy and impaired IC, research with results similar to ours explains this relationship, specifically attributing it to the cognitive domain of IC^20^. Higher maternal and paternal educational attainment is associated with a slower rate of cognitive decline in their children’s middle age and old age, possibly mediated by more stimulating family environments and better childhood health care^19,22^.

Food insecurity in childhood, characterized by reduced or missed meals and the substitution of nutritious foods with cheaper, nutrient-poor alternatives, can lead to insufficient intake of essential nutrients, compromising physical, cognitive, and psychosocial development. Nutritional deprivation in childhood has been consistently linked to cognitive decline in later life^23^, to greater functional limitations, to poorer lung function^24^, and to reduced sensory capacity (impaired vision and hearing development)^13^. Evidence of food insecurity among older adults has shown that the constant search for food leads to ongoing worry, mental distress, and chronic stress, which can affect psychological well-being by increasing the risk of depression, depressive symptoms, and cognitive impairment^25^.

In Brazil, access to piped water during childhood is a significant marker of inequities in access to essential services throughout the life course. Despite expanded coverage, inequalities in water supply and limitations regarding water quality persist, particularly affecting low-income populations and rural areas, with more than 40 million Brazilians still lacking adequate access^26^. Although its importance as a health determinant is recognized, there is a scarcity of studies that assess its association with IC in isolation. International evidence indicates that early-life exposures related to water and sanitation influence outcomes in old age, including poorer cognitive performance and markers of accelerated cellular aging^27^.

Regarding variables from adulthood, both educational attainment and income had a direct effect on IC and mediated the effects of childhood conditions. Education, in addition to mediating adult income, emerged as the primary determinant of IC in older adults and the elderly, indicating that early exposure to education may mitigate adverse effects throughout life and promote better health in old age.

Previous studies show similar results: Huang et al.^12^ observed that upward social mobility—an improvement in socioeconomic status over the course of one’s life—is associated with better IC in old age. Complementarily, another study found that individuals who grew up in unfavorable socioeconomic contexts but attained a high level of education in adulthood had an active life expectancy similar to that of those from economically privileged homes, highlighting the protective role of education in mitigating early-life disadvantages^28^.

A study of Brazilian older adults showed that unfavorable socioeconomic conditions in childhood are linked to poorer cognitive performance in old age. However, socioeconomic status in adulthood was the factor that explained this relationship: it fully mediated the effect on memory and partially mediated the effect on verbal fluency^20^.

According to the cognitive reserve theory, cognitive abilities accumulated over the course of a lifetime—such as those resulting from the level of education attained by early adulthood—persist, at least in part, even as one ages and strengthen the neurobiological reserve that promotes greater resistance to cognitive and functional decline in later life^29^.

In this context, favorable socioeconomic conditions—such as higher levels of education—expand access to professions that require complex cognitive skills and offer better-paying job opportunities, which in turn ensure greater participation in leisure activities, social engagement, and regular physical activity. These factors act synergistically, which appear to strengthen the development and maintenance of cognitive reserve throughout life^30^, thereby optimizing IC. Considering these findings and given the growing elderly population and rising educational levels among more recent generations, education may positively influence IC.

Overall, the results contribute to the gerontological literature by demonstrating that adverse socioeconomic conditions in childhood remain associated with impaired IC six to seven decades later, reinforcing the relevance of the life-course perspective in the study of aging. Education also stands out as a central axis in these relationships, acting both as a direct protective factor and as a mediator of the effects of early disadvantages. By quantifying these mediational pathways, the study advances our understanding of the mechanisms through which early inequities accumulate and are perpetuated throughout life.

Taken together, these findings broaden our understanding of the determinants of healthy aging and reinforce the potential of interventions that act early and continuously throughout the life course^6,7^. In this regard, the results point to the need for public policies that integrate actions aimed at improving conditions in childhood, strengthening education, and expanding socioeconomic opportunities in adulthood. Such an approach promotes a comprehensive perspective on aging, aligned with the needs of older adults and the organization of health care systems based on integrated, person-centered care.

This study has some limitations. First, the cross-sectional design of the study prevents the establishment of causal relationships between the variables. Although the associations found are statistically significant, data collection at a single point in time does not allow for determining the temporal precedence of events. Additionally, the study relied on retrospective measures of life course, which introduces potential memory bias. Reliance on self-reports for events that occurred several decades ago may be subject to recall bias or a subjective reinterpretation of past experiences, influenced by the participants’ current health status. Although the use of validated life course instruments and questions aims to minimize such distortions, the subjective nature of retrospective reporting remains a limiting factor in the accuracy of the magnitude of these events. Regarding data analysis, it should be noted that the models were estimated without applying sample weights. As a strategy to reduce potential violations of assumptions (particularly those related to heteroscedasticity), robust standard errors (vce robust) were used. It should also be noted that, although ELSI-Brasil is a representative survey of Brazilian adults and older adults, the variables used in the analyses had approximately 40% missing data; however, there were no statistically significant differences between the included and excluded groups for the life course variables. Despite these limitations, path analysis is a robust strategy, as it allows for the simultaneous identification of direct and indirect effects between variables, representing an advantage over traditional regression analyses.

In conclusion, it was evident that adverse childhood conditions related to a low socioeconomic status (parental illiteracy, lack of treated water, and food insecurity) are associated with impaired IC. This association manifests itself both through a direct effect of these adversities and through an indirect effect, mediated by life trajectories characterized by lower educational attainment and income in adulthood.
