# Supplementary material for: Life course and intrinsic ability in Brazilian adults and older adults: a mediation analysis
Source: Rev Saude Publica. 2026 Oct 19;60:e47. doi: 10.11606/s1518-8787.2026060007512 (PMC13719149; doi:10.11606/s1518-8787.2026060007512)
Supplement: Translation

# Translation: Curso de vida e capacidade intrínseca em adultos e idosos brasileiros: análise de mediação

**Authors:** Milara Barp Dias, Flávia Silva Arbex Borim, Daniella Pires Nunes, Isabella Amorim de Araújo Pereira, Valéria Pagotto

**Affiliations:** I Brasil Universidade Federal de Goiás. Programa de Pós-Graduação em Enfermagem e Saúde. Goiânia, GO, Brasil; II Brasil Universidade Estadual de Campinas. Programa de Pós-Graduação em Gerontologia. Campinas, SP, Brasil; III Brasil Universidade Cidade de São Paulo. Programa de Pós-Graduação em Fisioterapia. São Paulo, SP, Brasil; IV Brasil Universidade Estadual de Campinas. Programa de Pós-Graduação em Enfermagem. Campinas, SP, Brasil

**Keywords:** Curso de Vida, Capacidade Intrínseca, Adultos, Idosos, Envelhecimento

## Abstract

**OBJETIVO:**

Analisar as associações diretas e indiretas entre condições adversas ao longo do curso de vida e o comprometimento da capacidade intrínseca em brasileiros com 50 anos ou mais.

**MÉTODOS:**

Estudo transversal analítico com dados da segunda onda do Estudo Longitudinal da Saúde dos Idosos Brasileiros (ELSI-Brasil) (n = 4.817). A capacidade intrínseca foi avaliada em cinco domínios (cognição, vitalidade, locomoção, aspectos psicológicos e sensoriais), considerando-se como comprometida quando três ou mais domínios foram afetados. As adversidades na infância incluíram analfabetismo parental, ausência de água encanada no domicílio e insegurança alimentar. Escolaridade e renda na vida adulta foram consideradas variáveis mediadoras. Utilizou-se a análise de caminhos (*path analysis*).

**RESULTADOS:**

As adversidades na infância apresentaram efeitos diretos e indiretos significativos sobre o comprometimento da capacidade intrínseca. Esses efeitos foram mediados por menor escolaridade e renda na vida adulta. A escolaridade destacou-se como mediadora central, exercendo efeito protetor direto sobre a capacidade intrínseca e efeito indireto por meio de sua influência sobre a renda.

**CONCLUSÕES:**

Os achados reforçam a relevância da perspectiva do curso de vida para a compreensão das desigualdades no envelhecimento e indicam a escolaridade como alvo prioritário de intervenções voltadas à promoção do envelhecimento saudável no contexto brasileiro.

## INTRODUÇÃO

A capacidade intrínseca (CI) é definida como o conjunto de atributos físicos e mentais de uma pessoa que interagem com o ambiente e que, quando mantidos e otimizados ao longo da vida, favorecem o envelhecimento saudável. Proposto em 2019 a partir do *English Longitudinal Study on Ageing* (ELSA), o conceito avalia cinco domínios: locomoção, vitalidade, cognição, aspectos psicológicos e sensoriais. Embora seja um pilar da “Década do Envelhecimento Saudável (2021–2030)”, as evidências sobre a CI no cenário brasileiro ainda são escassas, especialmente no que tange à compreensão de como as trajetórias de vida no Brasil, marcadas por desigualdades históricas, impactam esses domínios de forma integrada. Essa lacuna limita a criação de estratégias específicas que considerem a heterogeneidade do envelhecimento no Brasil^1^.

Essa abordagem abrangente da saúde dos idosos reflete um avanço conceitual importante diante da constatação de que concepções de envelhecimento centradas exclusivamente em doenças ou incapacidades são insuficientes para representar a complexidade de vida e saúde das pessoas em seu processo de envelhecimento^1^. Enquanto a funcionalidade foca, em termos gerais, no que a pessoa *consegue fazer* (como realizar atividades da vida diária), a CI foca no que a pessoa tem em termos de reservas e potenciais biológicos e psicológicos. Estudos prévios identificaram fatores sociodemográficos e de saúde associados à CI, incluindo sexo^2^, idade^3^, nível de renda^4^, situação conjugal^4^, nível de escolaridade^5^, comportamentos relacionados à saúde^5^ e carga de doenças crônicas^3,4^.

Apesar da relevância desses estudos, análises realizadas em um único momento da vida podem não capturar plenamente os fatores que contribuem para a ocorrência da CI, tendo em vista que a trajetória de vida de cada pessoa varia ao longo do tempo e é influenciada por fatores individuais, sociais e contextuais.

Diante disso, estudar a CI a partir da perspectiva do curso de vida é essencial para compreender como experiências, exposições e contextos vividos ao longo da trajetória individual influenciam o envelhecimento e a saúde em idades avançadas, considerando que o envelhecimento é um processo multideterminado, contínuo e dinâmico. Os modelos do curso de vida teorizam que eventos em períodos críticos do desenvolvimento humano e o acúmulo de riscos ao longo da vida influenciam a saúde, as doenças e o envelhecimento^6,7^. Adversidades na infância, como baixa condição socioeconômica^8^ e insegurança alimentar^9^, estão associadas ao desenvolvimento de doenças físicas^10^ e transtornos mentais na vida adulta^11^.

A relação entre variáveis de trajetória de vida e CI já foi documentada^12,13^. Situações da infância como a baixa escolaridade parental, pior saúde na infância e residência em domicílios precários tiveram associação direta com menores níveis de CI em idades mais avançadas, com parte desse efeito mediado por desigualdades socioeconômicas acumuladas^13^. Além disso, os domínios da CI mais afetados por essas condições são principalmente o cognitivo, o sensorial e o psicológico^12^.

Evidências sobre a associação entre trajetórias do curso de vida e CI comprometida permanecem escassas no contexto brasileiro, particularmente considerando todos os seus domínios. Além disso, esse enfoque possibilita o avanço do conhecimento científico, integrando perspectivas biológicas, psicológicas e sociais, o que enriquece a compreensão sobre os processos de envelhecimento.

Frente a isso, o objetivo do estudo foi analisar a associação direta e indireta de condições adversas do curso de vida na CI comprometida em uma população brasileira de adultos com 50 anos ou mais, utilizando dados da linha de base do Estudo Longitudinal da Saúde dos Idosos Brasileiros (ELSI-Brasil).

## MÉTODOS

### Desenho do Estudo e População

Estudo transversal e analítico, baseado em dados da segunda onda do ELSI-Brasil, realizado entre 2019 e 2021 (Figura 1). O ELSI-Brasil é uma coorte nacional, de base domiciliar, representativa da população brasileira com 50 anos ou mais, residente na comunidade, cujo objetivo é investigar o processo de envelhecimento e seus determinantes, subsidiando a formulação e a avaliação de políticas públicas no país^14^.

**Figura 1:** Fluxograma de síntese da amostragem. ELSI-Brasil 2019–2021.

### Amostra e Amostragem do Estudo

O plano amostral do ELSI-Brasil é complexo, probabilístico e em múltiplos estágios, incluindo a seleção de municípios, setores censitários e domicílios. O desenho garante representatividade nacional, contemplando áreas urbanas e rurais de 70 municípios distribuídos nas cinco regiões brasileiras (Figura 1).

A linha de base do estudo foi conduzida entre 2015 e 2016, com 9.412 participantes. Na segunda onda, 6.172 indivíduos foram reentrevistados, ocorreram 2.270 perdas de seguimento e 970 óbitos. Para manter a representatividade etária da coorte, foram incluídos 3.777 novos participantes, totalizando 9.949 indivíduos entrevistados na segunda onda^14^ (Figura 1).

Para o presente estudo, foram utilizados apenas dados transversais da segunda onda. Todos os residentes dos domicílios selecionados com idade igual ou superior a 50 anos foram elegíveis. A amostra analítica final foi composta por 4.817 indivíduos, incluindo apenas os participantes com informações completas para todas as variáveis de interesse (Figura 1).

### Coleta de Dados

A coleta de dados foi realizada por entrevistadores previamente treinados, por meio da aplicação de questionários padronizados, além da realização de testes físicos e exames laboratoriais, seguindo protocolos previamente descritos no estudo ELSI-Brasil^14^.

### Variáveis do Estudo

#### Variável dependente

A variável de interesse foi a CI avaliada com base em cinco domínios: cognição, saúde psicológica, sensorial, vitalidade e locomoção^1^.

O domínio cognitivo foi mensurado pelo Teste de Fluência Verbal (TFV), no qual os participantes nomearam o maior número possível de animais em um minuto. Os pontos de corte foram de nove palavras para indivíduos com até oito anos de escolaridade e 13 para os demais, indicando cognição preservada^15^.

O domínio psicológico foi avaliado pela escala reduzida CES-D, com oito itens sobre sintomas depressivos. Escores ≤ 4 indicaram ausência de comprometimento^16^.

A vitalidade foi medida pela força de preensão manual com dinamômetro, utilizando a média de três tentativas. Os pontos de corte foram > 27 kgf para homens e > 16 kgf para mulheres, conforme critérios do *European Working Group on Sarcopenia*^17^.

A locomoção foi avaliada pelo teste de caminhada habitual em percurso de três metros, avaliada em duas medidas. Valores médios ≥ 0,8 m/s indicaram preservação do domínio^18^.

A avaliação sensorial considerou autorrelato da capacidade visual e auditiva. Respostas “muito boa”, “boa” ou “regular” para visão de longe, de perto e audição indicaram domínio preservado^3^.

Cada domínio da CI foi classificado como 0 (preservado) ou 1 (comprometido). A soma desses escores gerou um índice variando de 0 a 5 pontos, sendo considerada CI comprometida a presença de três ou mais domínios comprometidos^2^.

#### Variáveis independentes

As variáveis independentes incluíram fatores sociodemográficos – sexo (feminino ou masculino, autodeclarado) e faixa etária (categorizada em 50–59, 60–69, 70–79 e ≥ 80 anos) – e variáveis do curso da vida, a saber: analfabetismo parental ( relato de pai e mãe analfabetos)^19^, insegurança alimentar (relato de que, desde o nascimento até os 15 anos de idade, alguma vez dormiu com fome ou presenciou falta de alimentos)^9^, ausência de água tratada (relato de que não havia água canalizada na casa onde morava aos 10 anos de idade)^20^– todas dicotomizadas como “sim” ou “não”. Entre as variáveis mediadoras na vida adulta, estão a escolaridade (anos completos de estudo, categorizados como: nenhum, 1–4, 5–8 ou ≥ 9 anos) e a renda mensal (expressa em salários-mínimos, com base no valor de R$ 1.047,67, e categorizada como: < 1, 1–3 ou ≥ 4 salários-mínimos)^13,20^.

## Análise de Dados

Foi realizada análise descritiva para a caracterização da amostra, a partir das medidas de frequência absoluta e relativa para as variáveis. Foram estimadas as distribuições percentuais e respectivos intervalos de confiança de 95%, utilizando-se o módulo *survey* (svy), incluindo pesos amostrais e estratificação. Já as análises de equações estruturais foram realizadas por meio do comando sem e erros-padrão robustos (vce robust).

Para estudar a relação entre as variáveis de interesse, com base no modelo teórico preestabelecido (Figura 2), foi utilizada a análise de equações estruturais por meio da técnica de análise de caminhos (*Path Analysis*). Essa abordagem funciona como uma extensão do modelo de regressão, permitindo avaliar as relações entre múltiplas variáveis^21^. Com essa técnica, foi possível investigar as conexões diretas e indiretas entre variáveis independentes e dependentes.

**Figura 2:** Modelo hipotético da relação da CI com condições adversas da trajetória de vida. CI: capacidade intrínseca.

Os efeitos diretos das condições da infância sobre a CI são indicados pelas setas que ligam diretamente as variáveis independentes à variável dependente. Os efeitos indiretos, mediados por escolaridade e renda, são representados pelas setas que passam pelas variáveis mediadoras (Figura 2).

Após o ajuste dos indicadores e a realização dos testes de significância, foi construído o modelo final de análise de trajetórias, que manteve ou eliminou as relações do modelo teórico inicial. Os seguintes testes e valores de referência para os limiares de ajuste do modelo foram adotados neste estudo: teste do qui-quadrado (χ^2^) para qualidade de ajuste (p > 0,05), razão qui-quadrado por graus de liberdade (χ^2^/gl) < 2, *Standardized Root Mean Square Residual* (SRMR) ≤ 0,10, *Root Mean Square Error of Approximation* (RMSEA) ≤ 0,08, *Comparative Fit Index* (CFI) ≥ 0,90 e *Tucker–Lewis Index* (TLI) ≥ 0,90.

Para avaliar a adequação dos caminhos propostos, foram realizados testes de significância dos coeficientes de trajetória. Valores absolutos de t > 1,96 foram considerados estatisticamente significativos. As análises foram conduzidas no *software* Stata, versão 15.1.

## Considerações Éticas

O estudo foi aprovado pelo Comitê de Ética da Fundação Oswaldo Cruz (Fiocruz Minas) (CAAE: 34649814.3.0000.5091), e todos os participantes assinaram o termo de consentimento.

## RESULTADOS

A amostra foi composta por 4.817 participantes. Do total de participantes (n = 4.817), 54,7% eram mulheres, 50,4% apresentavam entre 50 e 59 anos, 37,9% com 1 a 4 anos de escolaridade. Quanto às variáveis de curso de vida, 43,7% dos idosos apresentavam pais analfabetos, 64,0% vivenciaram ausência de água encanada na infância e 24% passaram por situação de insegurança alimentar (Tabela 1).

**Tabela 1:** Características dos participantes do estudo (n = 4.817).

Variável	Total (n = 4.817)	IC95%
Sexo		
Feminino	54,7	51,7 a 57,7
Masculino	45,3	42,2 a 48,3
Faixa etária		
50–59 anos	50,4	46,0 a 54,4
60–69 anos	30,2	28,6 a 32,1
70–79 anos	14,7	12,6 a 17,1
≥ 80 anos	4,7	3,7 a 5,9
Escolaridade (anos de estudo)		
Nunca estudou	11,1	8,4 a 14,6
1–4	37,9	34,7 a 41,1
5–8	21,3	19,2 a 23,4
≥ 9	29,8	26,1 a 33,6
Renda (salários-mínimos)		
< 1	64,3	60,2 a 68,1
1–3	26,2	23,3 a 29,3
≥ 4	9,6	7,6 a 11,9
Analfabetismo dos pais na infância		
Não	56,3	50,9 a 61,6
Sim	43,7	38,4 a 49,1
Ausência água encanada na infância		
Não	36,0	31,4 a 40,7
Sim	64,0	59,3 a 68,5
Insegurança alimentar na infância		
Não	76,0	72,5 a 79,3
Sim	24,0	20,7 a 27,5
Capacidade intrínseca comprometida		
Não	73,3	68,3 a 77,7
Sim	26,7	22,3 a 31,7

IC95%: intervalo de confiança de 95%.

A Figura 3 mostra que tanto as variáveis de início da vida (analfabetismo parental, a ausência de água encanada e a insegurança alimentar) como as variáveis da vida adulta (renda e escolaridade) apresentaram associação direta com o comprometimento da CI. Observou-se que variáveis do início da vida, como analfabetismo parental (β = 0,07), ausência de água encanada (β = 0,03) e insegurança alimentar (β = 0,05), apresentaram associações diretas positivas com o comprometimento da CI. Por outro lado, maior escolaridade (β = -0,14) e maior renda (β = -0,04) mostraram efeitos diretos protetores. As condições adversas na infância também apresentaram associações negativas com a escolaridade (analfabetismo parental: β = -0,36; ausência de água encanada: β = -0,24; insegurança alimentar: β = -0,06), que, por sua vez, esteve positivamente associada à renda na vida adulta (β = 0,19). Adicionalmente, observou-se que renda e escolaridade mediaram a associação entre as condições do início da vida e a CI. Destaca-se que a escolaridade também atuou como mediadora da associação entre renda e CI, evidenciada pelo efeito indireto significativo (β = -0,01; p = 0,009) (Figura 3).

**Figura 3:** Modelo final da relação das variáveis de curso de vida com a CI comprometida, segundo *Path Analysis*. CI: capacidade intrínseca.

Nota: as setas representam associações direcionais estimadas no modelo, e os valores correspondem aos coeficientes padronizados (β). Setas mais espessas indicam associações diretas.

A Tabela 2 apresenta os efeitos indiretos, evidenciando que todas as condições da infância (analfabetismo parental: β = 0,06; ausência de água encanada: β = 0,04; insegurança alimentar: β = 0,01) influenciaram significativamente o comprometimento da CI na vida adulta, principalmente por meio de menores níveis de escolaridade e renda. Em relação às variáveis da vida adulta, destaca-se que somente a escolaridade apresentou efeito indireto protetor na CI mediado pela maior renda na vida adulta (Tabela 2).

**Tabela 2:** Efeitos indiretos mediados por escolaridade e renda sobre a capacidade intrínseca comprometida.

Associações indiretas	Variável mediadora	Coef.	p > |z|	IC95%
Ausência de água encanada → CI comprometida	Renda	0,0368	< 0,001	0,0286 a 0,0450
Insegurança alimentar → CI comprometida	Renda	0,0117	< 0,001	0,0068 a 0,0166
Escolaridade → CI comprometida	Renda	-0,0051	0,009	-0,0089 a -0,0013
Analfabetismo parental → CI comprometida	Escolaridade	-0,0489	< 0,001	-0,0684 a -0,0293
Ausência de água encanada → CI comprometida	Escolaridade	-0,0039	0,154	-0,0092 a 0,0014
Insegurança alimentar → CI comprometida	Escolaridade	-0,0251	0,034	-0,0482 a -0,0019
Analfabetismo parental → CI comprometida	Renda	0,0521	< 0,001	0,0411 a 0,0631

Coef.: coeficientes padronizados; IC95%: intervalo de confiança de 95%; CI: capacidade intrínseca.

O modelo final de equações estruturais demonstrou adequado ajuste aos dados, conforme indicado pelos parâmetros descritos na análise de dados (CFI =1,00; TLI = 1,00; RMSEA < 0,001; SRMR < 0,001), indicando boa adequação da estrutura proposta. No conjunto, o modelo explicou 26,1% da variação do comprometimento da CI.

## DISCUSSÃO

Os resultados deste estudo mostraram que condições indicativas de baixo nível socioeconômico na infância – analfabetismo parental, ausência de água encanada e insegurança alimentar – mostraram associação significativa com o comprometimento da CI, efeito que ocorreu tanto de forma direta quanto por meio da mediação de menor escolaridade e renda.

Embora a avaliação do curso de vida seja objeto de investigação de muitas pesquisas, sua relação com a CI foi avaliada em alguns estudos recentes^12,13^. Em uma análise geral sobre o efeito de várias condições da infância nos domínios da CI de pessoas adultas e idosos, dois estudos mostram achados semelhantes ao nosso. Um estudo transversal na China com pessoas ≥ 45 anos mostrou que baixa escolaridade parental, pior saúde na infância e condições domiciliares precárias associaram-se a menor CI, com parte desse efeito mediado por desigualdades socioeconômicas acumuladas ao longo do curso de vida. As associações mais fortes foram identificadas nos domínios cognitivo, sensorial e psicológico da CI^13^. Em um estudo com abordagem observacional baseada em coortes com participantes ≥ 50 anos dos Estados Unidos, Reino Unido, Europa, China e Japão, os níveis mais elevados de educação parental, condição socioeconômica na infância e renda familiar na vida adulta mostraram-se positivamente associados à CI em todas as coortes analisadas^12^.

Em relação ao analfabetismo parental e ao comprometimento da CI, há pesquisa com resultados semelhantes aos nossos que explica essa relação, especificamente atribuindo ao domínio cognitivo da CI^20^. Maior escolaridade materna e paterna associa-se a um declínio cognitivo mais lento na meia-idade e na velhice dos filhos, possivelmente mediado por ambientes familiares mais estimulantes e melhores cuidados em saúde na infância^19,22^.

A insegurança alimentar na infância, marcada pela redução ou ausência de refeições e pela substituição de alimentos nutritivos por opções mais baratas e pobres em nutrientes, pode levar à ingestão insuficiente de nutrientes essenciais, comprometendo o desenvolvimento físico, cognitivo e psicossocial. A privação nutricional na infância esteve consistentemente relacionada ao declínio cognitivo em idades mais avançadas^23^, a maiores limitações funcionais, a um pior desempenho da função pulmonar^24^ e à baixa capacidade sensorial (comprometimento no desenvolvimento da visão e audição)^13^. Evidência da insegurança alimentar em população de adultos mais velhos, mostrou que a busca constante por alimentação gera preocupação contínua, sofrimento mental e estresse crônico, o que pode afetar o domínio psicológico, a partir do aumento do risco de depressão, sintomas depressivos e comprometimento cognitivo^25^.

No Brasil, a disponibilidade de água encanada na infância constitui um marcador relevante das iniquidades no acesso a serviços essenciais ao longo do curso de vida. Apesar da ampliação da cobertura, persistem desigualdades no abastecimento e limitações quanto à qualidade da água, afetando especialmente populações de baixa renda e áreas rurais, com mais de 40 milhões de brasileiros ainda sem acesso adequado^26^. Embora sua importância como determinante de saúde seja reconhecida, há escassez de estudos que avaliem isoladamente sua associação com a CI. Evidências internacionais indicam que exposições precoces relacionadas à água e ao saneamento influenciam desfechos na velhice, incluindo pior desempenho cognitivo e marcadores de envelhecimento celular acelerado^27^.

Em relação às variáveis da vida adulta, tanto a escolaridade quanto a renda tiveram efeito direto sobre a CI e mediaram os efeitos das condições de infância. A escolaridade, além de mediar a renda na vida adulta, destacou-se como o principal determinante da CI em adultos mais velhos e idosos, indicando que a exposição precoce à educação pode atenuar efeitos adversos ao longo da vida e favorecer melhores condições de saúde na velhice.

Estudos anteriores mostram resultados semelhantes: Huang et al.^12^ observaram que a mobilidade social ascendente – melhoria no *status* socioeconômico ao longo da vida – está associada a melhor CI em idade avançada. De forma complementar, outro estudo verificou que indivíduos que cresceram em contextos socioeconômicos desfavoráveis, mas alcançaram elevada escolaridade na vida adulta, apresentaram expectativa de vida ativa semelhante à daqueles oriundos de lares economicamente privilegiados, evidenciando o papel protetor da educação na mitigação das desvantagens precoces^28^.

Um estudo com idosos brasileiros mostrou que condições socioeconômicas desfavoráveis na infância estão ligadas a um pior desempenho cognitivo quando idosos. No entanto, a situação socioeconômica na idade adulta foi o fator que explicou essa relação: ela mediou completamente o efeito na memória e mediou parcialmente o efeito na fluência verbal^20^.

Conforme a teoria da reserva cognitiva, habilidades cognitivas acumuladas ao longo da vida – como proporcionadas pelo nível de escolaridade adquirido até o início da vida adulta – persistem, pelo menos em parte, mesmo com o avançar dos anos e fortalecem a reserva neurobiológica que promove maior resistência a declínios cognitivos e funcionais nas fases mais avançadas da vida^29^.

Nesse contexto, condições socioeconômicas favoráveis – como maior escolaridade – ampliam o acesso a profissões que exigem habilidades cognitivas complexas e oferecem oportunidades de trabalho com melhor remuneração, que garantem maior participação em atividades de lazer, engajamento social e prática regular de atividade física. Esses fatores atuam de forma sinérgica, o que parece fortalecer a construção e manutenção da reserva cognitiva ao longo da vida^30^, otimizando a CI. Considerando esses achados, e diante do aumento da população idosa e da elevação do nível educacional nas gerações mais recentes, a educação poderá influenciar positivamente a CI.

De modo geral, os resultados contribuem para a literatura gerontológica ao evidenciar que condições socioeconômicas adversas na infância permanecem associadas ao comprometimento da CI seis a sete décadas depois, reforçando a relevância da perspectiva do curso de vida no estudo do envelhecimento. Destaca-se ainda a escolaridade como eixo central dessas relações, atuando tanto como fator protetor direto quanto como mediadora dos efeitos das desvantagens precoces. Ao quantificar esses caminhos mediacionais, o estudo avança na compreensão dos mecanismos pelos quais iniquidades iniciais se acumulam e se perpetuam ao longo da vida.

Em conjunto, esses achados ampliam o entendimento sobre os determinantes do envelhecimento saudável e reforçam o potencial de intervenções que atuem precocemente e de forma contínua ao longo do curso de vida^6,7^. Nesse sentido, os resultados apontam para a necessidade de políticas públicas que integrem ações voltadas à melhoria das condições na infância, ao fortalecimento da educação e à ampliação de oportunidades socioeconômicas na vida adulta. Tal abordagem favorece uma perspectiva integral do envelhecimento, alinhada às necessidades das pessoas idosas e à organização de sistemas de saúde baseados no cuidado integrado e centrado na pessoa.

Este estudo apresenta algumas limitações. Primeiramente, o delineamento transversal da pesquisa impede o estabelecimento de relações de causalidade entre as variáveis. Embora as associações encontradas sejam estatisticamente significativas, a coleta de dados em um único momento no tempo não permite determinar a precedência temporal dos eventos. Adicionalmente, a investigação baseou-se em medidas retrospectivas de curso de vida, o que introduz um potencial viés de memória. A dependência do autorrelato para eventos ocorridos há várias décadas pode estar sujeita a falhas de evocação ou a uma reinterpretação subjetiva das experiências passadas, influenciada pelo estado de saúde atual dos participantes. Embora o uso de instrumentos e questões de curso de vida validados busque minimizar tais distorções, a natureza subjetiva do relato retrospectivo permanece um fator limitante na precisão da magnitude desses eventos. Em relação à análise dos dados, destaca-se que os modelos foram estimados sem a aplicação de ponderações amostrais. Como estratégia para reduzir possíveis violações de pressupostos (particularmente relacionadas à heterocedasticidade), foram utilizados erros-padrão robustos (vce robust). Destaca-se, ainda, que, embora o ELSI-Brasil seja um inquérito representativo de adultos e idosos brasileiros, as variáveis utilizadas nas análises apresentaram cerca de 40% de informações ausentes, porém sem diferenças estatisticamente significantes entre o grupo incluído/excluído para as variáveis de curso de vida. Apesar dessas limitações, a análise de caminhos (*Path Analysis*) constitui uma estratégia robusta, ao permitir a identificação simultânea de efeitos diretos e indiretos entre variáveis, representando uma vantagem em relação a análises de regressão tradicionais.

Concluindo, evidenciou-se que condições adversas na infância, relacionadas a um baixo nível socioeconômico (analfabetismo parental, ausência de água tratada e insegurança alimentar), estão associadas ao comprometimento da CI. Essa associação se manifesta tanto por um efeito direto dessas adversidades quanto por um efeito indireto, mediado por trajetórias de vida caracterizadas por menor escolaridade e renda na idade adulta.
